# Multifunctional Nanomaterials: Synthesis, Properties, and Applications 2.0

**DOI:** 10.3390/ijms24087619

**Published:** 2023-04-21

**Authors:** Raghvendra Singh Yadav

**Affiliations:** Centre of Polymer Systems, Tomas Bata University in Zlin, Trida Tomase Bati 5678, 76001 Zlin, Czech Republic; yadav@utb.cz; Tel.: +420-576031725

This Special Issue, “Multifunctional Nanomaterials: Synthesis, Properties and Applications 2.0”, is composed of twelve published research articles, which include nine original research articles and three review articles.

An innovative method described by Zijun Yan et al. [1] was used to improve the efficiency of electrochemical CO_2_ reduction (CO_2_R) as well as the ability to produce C_2_ products. A simple hydrothermal process combined with calcination was used to create CuO/g-C_3_N_4_ based on copper oxide catalysts, using g-C_3_N_4_ as a carrier. The addition of g-C_3_N_4_ boosted the specific surface area, which promoted the kinetics of mass transfer and created new opportunities for the exposure of active sites and CO_2_ adsorption. Veloso, S.R.S. et al. [2] reported calcium-doped manganese ferrite nanoparticles via an oxidative precipitation approach for magnetic hyperthermia. The produced calcium-doped manganese ferrite nanoparticles had larger saturation magnetization and heating efficiency values, which made them suitable for therapeutic uses such as drug delivery and cancer treatment. Maraming, P. et al. [3] discussed the creation of an electrochemical aptasensor functionalized with polydopamine nanoparticles (PDA-NPs) for the rapid, accurate, and economical detection of glycated albumin (GA), a promising biomarker for glycemic control in diabetic patients. This suggested method demonstrated its potential use in GA measurement to improve diabetic patient screening and management in the future. Chen, D.R. et al. [4] reported a fabrication of a Zn-CO_2_ battery using a carbon nanotube (CNT) sheet as a cathode and a Zn plate as an anode. This study emphasized the significance of the activation process, which made it possible to load more catalyst onto the cathode and create more active sites for the electroreduction process. In order to design TiO_2_-Bi_2_S_3_ composites with high photoactive performance, a detailed vulcanization-process-dependent microstructure evolution and photoactive properties of TiO_2_-Bi_2_S_3_ composites were proposed in the study of Liang, Y.-C. et al. [5]. Furthermore, Zhu, P. et al. [6] used commercial microcrystalline cellulose (MCC) as the raw material to develop spherical cellulose nanocrystal (CNC) via mixed acid hydrolysis. By choosing different pretreatment routes through mixed acid hydrolysis, the authors showed that it is possible to prepare spherical CNC with various properties economically from MCC. The fabrication of anisotropic silver nanostars (AgNS) that can be used as highly effective surface enhanced Raman spectroscopy (SERS) substrates for various bioanalytes, even in the case of a near-infrared (NIR) excitation laser, was described by Revnic, R.N. et al. [7]. In order to neutralize V. *vulnificus* hemolysin (VvhA), Zou, S. et al. [8] created a biocompatible nanoscale detoxification system. With regard to recombinant V. *vulnificus* hemolysin (rVvhA)-induced toxicity, nanosponges (NSs) demonstrated excellent protective effects, offering helpful insights into how to counter the growing dangers posed by severe V. *vulnificus* infections. For the purpose of detecting different lung cancer cell types in hydroplegia, Mukundan et al. [9] reported the optical and material properties of a MoS_2_/Cu_2_O sensor.

Halloysite nanotubes, their properties, and their use in the biological field were reviewed by Biddeci, G. et al. [10]. Recent developments in metasurfaces for photocatalysis, surface-enhanced infrared absorption (SEIRA), and surface-enhanced Raman scattering (SERS) sensors were reported by Barbillon, G. [11]. The applications of magnetic resonance imaging (MRI), single-photon emission computed tomography (SPECT), and positron emission tomography (PET) in reporter gene technologies for use in brain imaging were discussed in a review article by Gao, T. et al. [12].
